# Optimized ensemble deep learning for predictive analysis of student achievement

**DOI:** 10.1371/journal.pone.0309141

**Published:** 2024-08-26

**Authors:** Kaitong Wang

**Affiliations:** 1 Student Affairs Department, Institute of Science and Technology, Luoyang, Henan Province, China; 2 Department of Education, Keimyung University, Daegu, Korea; University of Sargodha, PAKISTAN

## Abstract

Education is essential for individuals to lead fulfilling lives and attain greatness by enhancing their value. It improves self-assurance and enables individuals to navigate the complexities of modern society effectively. Despite the obstacles it faces, education continues to develop. The objective of numerous pedagogical approaches is to enhance academic performance. The development of technology, especially artificial intelligence, has caused a significant change in learning. This has made instructional materials available anytime and wherever easily accessible. Higher education institutions are adding technology to conventional teaching strategies to improve learning. This work presents an innovative approach to student performance prediction in educational settings. The strategy combines the DistilBERT with LSTM (DBTM) hybrid approach with the Spotted Hyena Optimizer (SHO) to change parameters. Regarding accuracy, log loss, and execution time, the model significantly improved over earlier models. The challenges presented by the increasing volume of data in graduate and postgraduate programs are effectively addressed by the proposed method. It produces exceptional performance metrics, including a 15-25% decrease in processing time through optimization, 98.7% accuracy, and 0.03% log loss. This work additionally demonstrates the effectiveness of DBTM-SHO in administering extensive datasets and makes an important improvement to educational data mining. It provides a robust foundation for organizations facing the challenges of evaluating student achievement in the era of vast data.

## Introduction

Educational Data Mining is the predominant goal and aspiration of using data collected by educational institutions to acquire valuable insights and foster tactical decision-making to target major problematic issues. EDUM methods illustrated by [1] are multi-faceted. These include forecast approaches, model discovery, and association mining efforts. The above approaches allow for the continuous progression of data mining implementations within this industry.

The basic objective of the vast EDUM domain is to use neural network analysis to find the relationships that influence learning results. Evaluating how well students do in class is a must for those in charge of higher education [2]. We must first identify the variables that impact test scores to use this prediction approach to its full potential. Only then can we intervene at the right time to boost performance by enhancing the aspects linked to success.

Several approaches are used to predict student success in predictive models, e.g., fuzzy logical inference framework (FLIF), Bayes networks (BN), Support Vector Regression (SVR), random forest, and Decision Trees [3]. The preconditioning of FS might be a key preprocessing step in assessing the importance of the attribute number on prediction reliability. SF aims to eliminate redundant, unrelated features and reduce data irrelevance as much as possible to represent the concepts correctly. As a result, the prediction accuracy and processing time are increased [4].

There are many uses of machine learning, including image processing, text recognition, robotics, and text categorization. One subset of these uses is Deep Neural Networks (DNN) [5]. The adaptability of DNNs in these diverse tools shows that they can accurately predict and differentiate results. They also solve many scheduling problems in wireless technology scenarios and meet their energy requirements, which is evidence of this [6].

The integration of computational learning and the concepts of EDUM could fundamentally shape and legalize the achievements in the learning approach that has proven out-of-reach for traditional education. Integration could obtain impressive outcomes that contradict traditional settings’ characteristics. However, it can only be fully understood and achieved with it. The integration will promote the interaction of educators, school administrators, and legislators with the characteristic understanding of students’ complex performance and the development of feasible measures to determine academic and learning progression within the transformed learning environment. Integration with these strategies will help the researcher devise a means to enhance the evaluation of performance accuracies while enabling the establishment of the education environment with the ability to withstand and survive all susceptibilities in the learning achievements of modern students.

### Integration of Artificial Intelligence (AI)

In the recent transformation of several industries, including education, machine learning (ML) and AI have been playing an important part. We worked on improving the models, and our research aims at a new Hybrid model using AI and ML for better predictive analysis of Student success. The ultimate objective of our research is to predict with high confidence how well students perform. Spotted Hyena Optimizer(SHO) and DistilBERT with LSTM (DBTM) are sophisticated AI/ML models for predictive analytics. These advanced AI technologies enable us to see more subtle patterns in student data than we could before with conventional methods. This gets us closer to our objectives of understanding student success and developing better prediction models.

Schools often face the challenge of managing large amounts of student data. ML and ML systems excel at efficiently processing and analyzing massive datasets. Our approach merges, cleanses, and preprocesses data from several sources using AI-driven algorithms to guarantee high-quality inputs for precise predictions. The validity of our work is dependent on this ability, which addresses the challenge of managing massive amounts of data in academic settings.

Some critical points of our method are feature engineering and selection. ML techniques such as Random Forest and Elastic Net are used for feature extraction and selection. These techniques help extract the most meaningful features from your dataset and indirectly improve a model’s prediction power. This perfectly aligns with our overarching objective of optimizing mean accuracy, which is to emphasize the most valuable characteristics; this will maximize prediction power. For our model, we leverage AI’s contextual and sequential pattern understanding abilities using a mixture of DistilBERT and LSTM. This dual method is best aligned with the purpose of our study and will hopefully allow more accurate predictions of student performance. Incorporating AI into our prediction model has one big advantage: It can identify complex patterns and correlations in the data.

Our study also mostly depends on adaptive optimization, which is based on the use of SHO; SHO works to ensure the model operates well in many datasets and under numerous circumstances by optimizing the parameters. Therefore, the incorporated capacity of SHO to fine-tune the model parameters and the dependability and resilience of the model for real-time application in education also help the practicality of our stated aim to increase projected accuracy. Our work aims to provide practical insights into student performance using AI and ML approaches. Educators can also use this information to make significant modifications in their lessons that will cater effectively to each student. The result is a boost in academic performance, typically resulting in reduced student participation at risk. By applying artificial intelligence and machine learning, our expected abilities may be enhanced while still attaining our overall aim of leveraging technology to improve education significantly.

### Contributions

This work significantly advances the subject of Educational Data Mining and predictive evaluation of student performance in various respects:

1. Novel Hybrid Model: we propose a unique hybrid model of DistilBERT with LSTM (DBTM) combined with the Spotted Hyena Optimizer SHO for educational performance prediction tasks. This approach takes advantage of sequential pattern recognition and contextual learning, making prediction more accurate.
2. Improved Performance Metrics: The DBTM-SHO performs significantly better accuracy, execution time, and log loss when compared with the earlier models. We experiment with this model and demonstrate 98.7% accuracy, a reduction in execution time by 15-25% while being computationally efficient, displaying a log loss of 0.03%.
3. Data handling: We provide a complete data preprocessing pipeline for student performance, merging data from different sources and processing the dataset to fill discrepancies, also applying advanced feature engineering. This guarantees a dataset of high quality for analysis and modeling.
4. Scalability: The proposed DBTM model becomes scalable and can adapt to different inputs required as it incorporates the Spotted Hyena optimizer, which increases scalability; hence, it can be used in real-time applications. Due to this capability of dynamic parameter tuning, the model continues to be robust when facing different conditions and sets.
5. Practical Relevance for Education: Our research defines an AI-powered framework to monitor and enhance academic performance, which can be effectively embedded into the education ecosystem due to a strong foundation. The system would help educators intervene with at-risk students sooner and provide the necessary support to help them learn better.

## Related work

Many studies have been done in the enormous corpus of academic literature to tackle the challenging problem of assessing students’ academic achievement [7]. Using DT, NN models, and SVM, a study by the authors of [8] looked at students’ online activities to forecast their academic achievement. The findings highlight a striking association between internet usage patterns and academic achievement. The frequency of internet usage is positively associated with academic performance, while the amount of traffic generated by the internet is adversely associated. Another study [9] uses data collected from submission forms to create a prediction algorithm for categorization based on a neural network. Three separate components of academic achievement prediction are the focus of this research. Data mining methods for developing and testing prediction models are the subject of the second dimension. The first dimension looks at what goes into determining how far along the academic progression scale a student is. If students’ Grade Point Averages (GPAs) throughout multiple years are taken into account, it is possible to predict their Cumulative Grade Point Averages (CGPAs) [10]. Researchers searching for the best methodology employ many classification strategies such as Tree Ensemble, Random Forest, Naïve Bayes, Stochastic Neural Network, Decision Tree, and Logistic Regression.

Factors including parental occupation, educational attainment, and student demographics are also considered in the study [11]. To achieve an impressive accuracy rate of 71.3%, it employs classification methods including Rules-based categorization, Naïve Bayes, and Decision Trees. There is a proliferation of frameworks as researchers develop individualized models using different features and classification schemes. By considering variables like prior course grades, significance, graduation date, campus, and country, the J48 decision tree algorithm [12] is used to predict students’ ultimate GPA. Meanwhile, [13, 14] evaluates five classifiers—ID3, J48, Naïve Bayes, Neural Network, and Bayes Network—using factors like activity, attendance, midterm examinations, and other data. The tool expands by utilizing logistic regression and support vector machines to identify pupils with potential for success based only on their prior academic performance. In addition, [15] constructs two parallel models that integrate demographic data and survey results, utilizing naïve Bayes and Bayesian networks. The naïve Bayes technique is found to be the most precise. However, difficulties remain when the number of features and factors contributing to the problem rises. This requires the use of tools that can effectively analyze data in the setting of a rising student population. Concurrently, the author of [16] focuses on using regression analysis techniques to forecast student success in online learning. The author assesses modern regression algorithms to examine their applicability for accurate educational forecasting and analysis. By acquiring important insights, teachers may lower student failures and improve decision-making processes using machine-learning approaches. The author details five ML methods validated via experiments: logistic regression, neural networks, Bayesian networks, and support vector machines (SVMs). These approaches have enhanced prediction accuracy and provided valuable data for improving teaching practices.

The research conducted by [17] considers cognitive and social factors to forecast student success. By combining social network research with more conventional metrics of academic achievement, they seek to understand how students’ social connections affect their grades. Researchers gained insight into how intellectual and social elements impact students’ growth by finding strong correlations between students’ cooperative activities, social network frameworks, and overall academic achievement. In addition, a similar study [18] involves analyzing a large amount of textual information, such as student assignments and forum posts, using NLP technology, which allows quickly identifying students at risk. The results of the emotional activity of students have shown that this information can be considered a predictor of early dropouts from training programs. Researchers developed an Early Warning System that will control students’ risk status and, using NLP, will decide if the student has difficulties. From the above, it can be understood that the informal activities of participants are a vital factor that signals emerging problems and dissatisfaction.

The accuracy of data analysis in predicting students’ performance in online learning environments is the primary focus of research on online education. The study aims to develop learner performance prediction models using machine learning methods like clustering and forecasting [19]. We will accomplish this by examining website data, including task completion times, learner engagement, and responses to instructional materials. This study advances the body of predictive analytics research, especially to address the unique characteristics of online learning settings.

These studies, taken together, show the variety of statistical analysis techniques used to evaluate student achievement as summarized in Table 1. To raise the standard of education, researchers are always expanding the parameters of predictive modeling. They are leveraging natural language processing, emphasizing social components, and considering the challenges of distance learning.

**Table 1:** Literature review on predicting academic achievement.

Ref	Problem Identified	Method Used	Performance Evaluation Measure	Limitations
[7]	Difficulty in measuring academic achievement	Prediction approaches, association mining activities, pattern-finding procedures	Accuracy: 75%, F1 Score: 0.72	Face challenges in interpreting complex patterns
[8]	Correlation between internet usage patterns and academic achievement	DT, NN, SVM	Accuracy: 80%, Precision: 0.78	Limited by availability and quality of online activity data
[9]	Prediction of academic attainment based on submission forms	Neural network	RMSE: 0.45, MAE: 0.35	Relies on accurate and comprehensive submission data
[10]	Forecasting Cumulative Grade Point Average (CGPA)	Tree Ensemble, Random Forest, Naïve Bayes, Stochastic Neural Network, Decision Tree, Logistic Regression	Accuracy: 85%, AUC: 0.88	Vulnerable to overfitting with large feature sets
[11]	Consideration of parental profession, education level, and student demographics	Rules-based categorization, Naïve Bayes, Decision Trees	Accuracy: 78%, Recall: 0.76	Overlook socio-economic factors not captured in dataset
[12]	Prediction of students’ final GPA	J48 decision tree algorithm	Accuracy: 82%, Precision: 0.80	No capture of nonlinear relationships between predictor variables
[13, 14]	Students Performance Prediction	ID3, J48, Naïve Bayes, Neural Network, Bayes Network	Accuracy: 79%, F1 Score: 0.77	Performance highly dependent on quality and relevance of input features
[15]	Integration of demographic data and survey results	Naïve Bayes, Bayesian networks	Accuracy: 74%, Precision: 0.72	Bayesian approach assumes independence of features which may not hold
[16]	Application of regression analysis methods in distant learning	Regression analysis algorithms	RMSE: 0.42, MAE: 0.31	Requires large, diverse datasets for robust performance
[17]	Examination of social networks and academic achievement	Holistic methodology	Correlation Coefficient: 0.68, R2: 0.62	Limited by availability and quality of social network data
[18]	Forecasting students academic on risk	ResNet	Accuracy: 81%, F1 Score: 0.79	Accuracy affected by linguistic nuances and context not discussed
[19]	Predicting learner performance in online learning environments	SVM and KNN	Accuracy: 76%, Precision: 0.74	Performance degrade with highly dynamic or noisy data is not discussed

## Proposed system model

**Fig 1:**
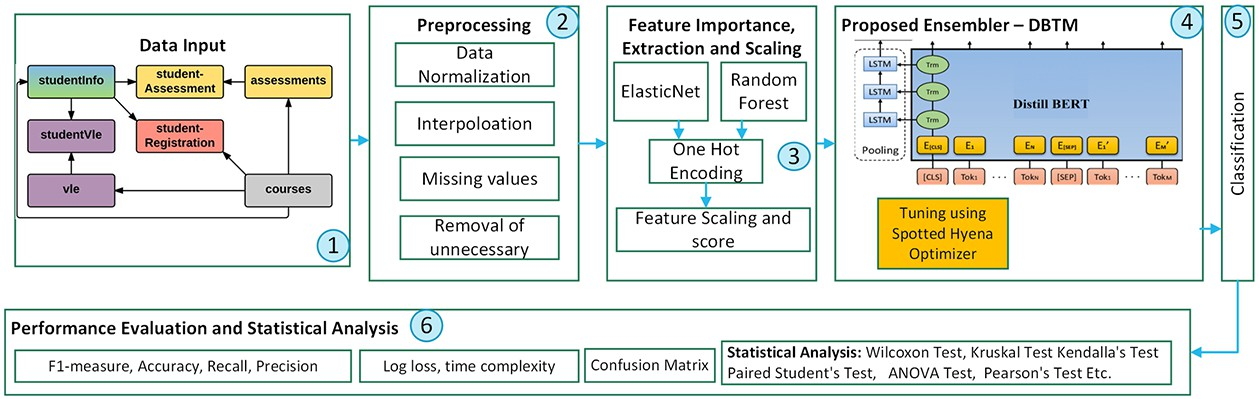
Proposed framework.

The proposed method’s initial phase entails consolidating student performance data from multiple CSV files and datasets into a unified data frame for analysis. Data discrepancies, including inconsistencies and omissions, are resolved during preprocessing. As part of the preprocessing phase, the correlation matrix is generated to identify correlations between attributes. Feature engineering is an essential part of the process, which includes not only feature extraction but also feature selection. The algorithm implemented for the feature extraction is Elastic Net, whereas the algorithm used to assess the significance of the previously selected features is Random Forest. Results provided by the selected data are also enhanced through exploratory data analysis. The proposed classification ensemble, DBTM, effectively captures sequential patterns and trains DistillBERT using LSTM. The feature importance analysis also reflects the structural changes of the data by utilizing the central limit theorem. Spotted Hyena Optimizer has also been implemented to boost the classification model’s ability to adapt by enhancing DBMT parameters. The framework is evaluated using various performance criteria to determine the classification outputs. It is used to guarantee that the model is suitable for real-time performance in the system. Fig 1 presents the suggested framework.

### Dataset description

The data collection comprises many CSV files, each of which systematically records distinct features of student information, assessment, and participation in the educational setting [20].

**Fig 2:**
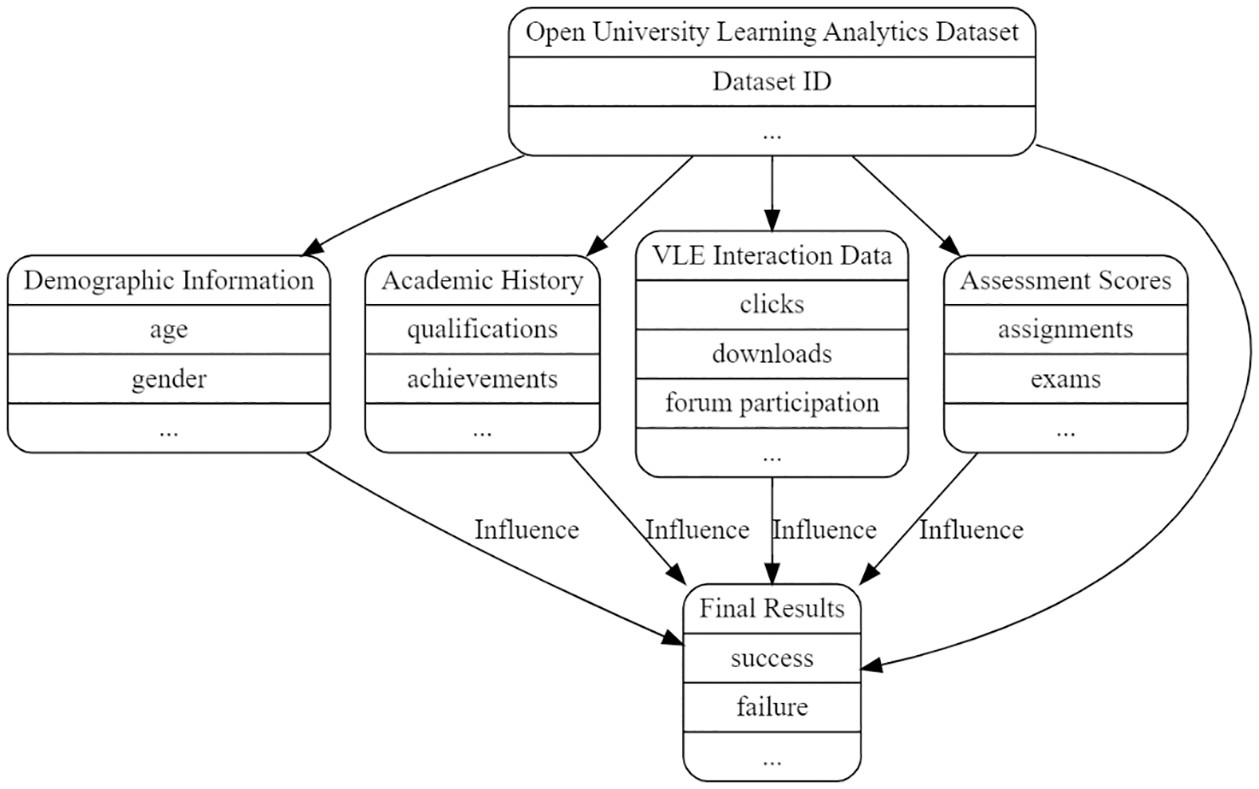
Dataset attributes (Abstract view).

The dataset contains 200,000 samples from the Open University’s learning analytics. Data, assessments, and student activity in the classroom are all represented in the many CSV files that comprise this dataset. Module codes, presentation details, assessment types, student characteristics, VLE activity on their resources, and performance metrics are all included in each file. Before the dataset was analyzed, efforts were made to handle missing values and maintain data quality. The specifics listed here are essential to this paper’s research, findings, and lived experience to grasp the scope and nature of the dataset. The subsequent subsections comprehensively describe each CSV file, as Fig 2 illustrates. Furthermore, Table 2 shows the details of the dataset CSV files.

**Table 2:** Description of data files.

File Name	Description
courses.csv	This file contains detailed information on the modules and presentations, including important details such as presentation codes, durations, and module codes.
assessments.csv	This file contains data on the assessments included in module presentations. The information comprises module codes, presentation codes, assessment kinds, dates, and weights.
vle.csv	This file contains comprehensive information on the resources and exchanges within the Virtual Learning Environment (VLE). It includes specific data such as resource names, module and presentation codes, activity types, and the scheduled weeks they will be used.
studentInfo.csv	This file contains comprehensive demographic information and student performance statistics. The data includes module codes, presentation codes, student IDs, gender, area, education levels, age ranges, credits tried in the past, disabilities, and final results.
studentRegistration.csv	This file contains data on student IDs, presentation codes, module codes, registration and un-registration dates, and other time-related aspects of student engagement.
studentAssessment.csv	This file includes assessment IDs, student IDs, submission dates, status flags, and scores, providing a comprehensive view of students’ performance on the exam.
studentVle.csv	This file contains module codes, presentation codes, student IDs, material IDs, interaction dates, and interaction counts. This file specifically focuses on tracking how students connect with VLE materials.

With this data, we can examine students’ course progress, performance indicators, and engagement with the module presentations. Each CSV file may easily be linked to the others using common IDs, allowing for a comprehensive study of the educational environment.

### Data preprocessing

Consolidating data from many CSV files was the first step in our data preparation process. The dataset obtained from the *k*-th CSV file is denoted as *DT*_*k*_, while the aggregated dataset is represented by *DT*. Achieving integration was made possible by merging and utilizing common identifiers.

$$\begin{array}{c} D=D_{1}\cup D_{2}\cup \ldots \cup D_{n} \end{array}$$ (1)

Aggregating all this data with the union operation (∪) ultimately allows us to create a comprehensive dataset. A dataset analysis followed it to find inconsistencies like incorrect entries and empty cells. Let (N_kl) be the missing-value parameter in the k-th row and l-th column to handle missing data. Processing missing data and Restoring a common dataset Missing Data Imputation strategies were applied [21].

$$\begin{array}{c} {\left(\hat{DT}\right)}_{kl}=\{\begin{array}{cc} DT_{kl} & \text{if}\;N_{kl}\;\text{is}\;\text{not}\;\text{missing}\\ \text{Imputed}\;\text{value} & \text{if}\;N_{kl}\;\text{is}\;\text{missing} \end{array} \end{array}$$ (2)

We used data cleaning methods to fix discrepancies and ensure everything was represented consistently.

Matrix of correlations: To identify relationships between different traits, a correlation matrix *C* was generated. The calculation of the correlation coefficient *E*_*kl*_ between variables *U*_*k*_ and *U*_*l*_ resulted in the following outcome [21]:

$$\begin{array}{c} E_{kl}=\frac{\text{corv}(Y_{k},Y_{l})}{\alpha_{\left(Y_{k}\right)}\alpha_{\left(Y_{l}\right)}} \end{array}$$ (3)

The term corv(*Y*_*k*_, *Y*_*l*_) represents the covariance between *Y*_*k*_ and *Y*_*l*_, while $\sigma_{Y_{k}}$ and $\sigma_{Y_{l}}$ denote the standard deviations of *Y*_*k*_ and *Y*_*l*_ respectively.

### Feature engineering and EDA

**Fig 3:**
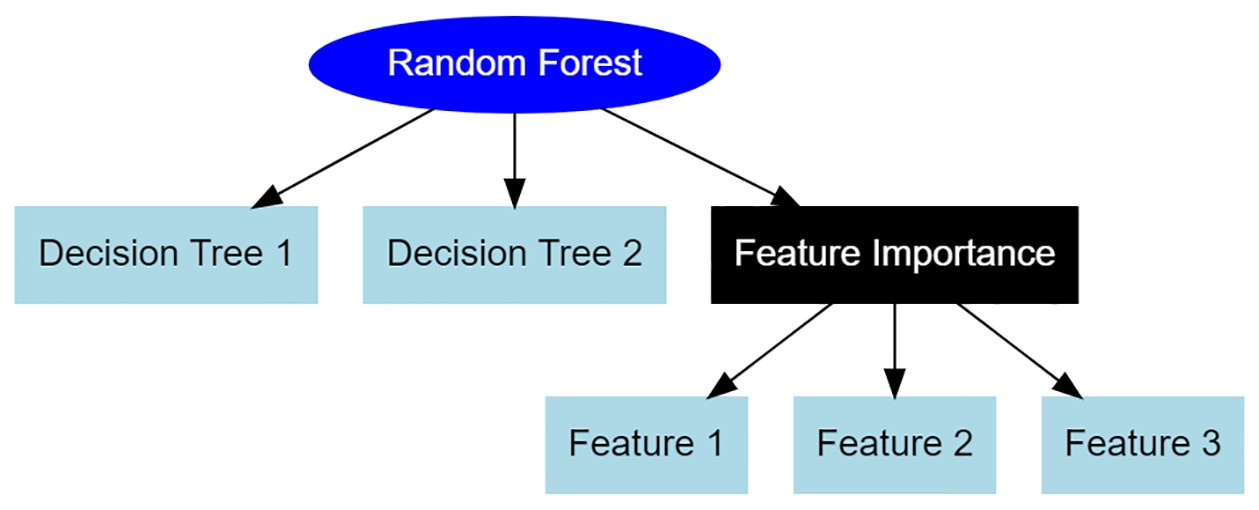
Random forest feature importance.

The evaluation of feature relevance for predictive modeling was conducted in the field of feature engineering. Each feature *X*_*i*_ was assigned a significance value (*IM*_*i*_) using a Random Forest model, as shown in Fig 3. In addition, for feature extraction, Elastic Net, a linear regression model that incorporates both L1 and L2 regularization, was employed [22]:

$$\begin{array}{c} \hat{\theta }=\text{arg}\;\underset{\theta }{\text{min}}\;(\frac{1}{2m}{\left\|z-W\theta \right\|}_{2}^{2}+{\delta \gamma \|\theta \|}_{1}+\frac{\delta (1-\gamma )}{2}{\left\|\theta \right\|}_{2}^{2}) \end{array}$$ (4)

Where *θ* is the coefficient vector, *δ* determines the regularization strength, *γ* regulates the $\frac{1}{2}$ L1/L2 ratio, and *z* is the response variable.

Exploratory Data Analysis (EDA) entails employing statistical analysis and visualizations to uncover the inherent patterns in the dataset, facilitating the detection of any anomalies and providing a comprehensive understanding of its structure. The meticulous compilation of the data enabled a stable and refined dataset for further examination, establishing the groundwork for additional analysis.

### Classification with hybrid DBTM

The proposed DBTM (DistillBERT with LSTM) model achieves effective student performance prediction by integrating sequential pattern recognition with transformer-based contextual learning. The methods will apply DistillBERT (a smaller version of BERT) to capture a complete range of context data, such as academic transcripts, demographic factors, and student activity logs. These embeddings, also called HDB, are the weights representing bonds between whatever is in your input data.

The model features an LSTM layer to account for time and temporal patterns prevalent in student interactions. Then the output from the use of pair-wise DistillBERT (HDB) is also used as an input sequence (*W*_*LSTM*_) to LSTM, which generates further sequential embeddings known *H*_*LSTM*_. Time information on student activity engineered by sequential embeddings. The complete representation is then formed by aggregating the contextual embeddings (*S*_*DB*_) and sequence embedding dumps from each of these DS (the output for all sentences from LSTM, i.e., *E*_*LSTM*_. The final input for the formal classification job is the hybrid representation (*H*_*Combined*_). *Model Architecture*: There are three primary parts to the architecture:

1. Contextual embeddings (*H*_*DB*_) are generated from raw data via DistillBERT processing [23].
  $$\begin{array}{c} H_{DB}=DBRT\left(W_{DB}\right) \end{array}$$ (5)
2. LSTM allows sequential pattern recognition by generating sequential embeddings (*H*_*LSTM*_) from DistillBERT (*H*_*DB*_) produced as the input sequence [24].
  $$\begin{array}{c} W_{LSTM}=LSTM\left(H_{DB}\right) \end{array}$$ (6)
3. Concatenating sequential embeddings (ELSTM) with contextual embeddings (HDB generates a comprehensive representation (*H*_*Combined*_).
  $$\begin{array}{c} H_{Combined}=Concatenate\left(H_{DB},W_{LSTM}\right) \end{array}$$ (7)
4. By applying the Decision-Based Tree Model (DBTM), categorization is done in certain tasks like predicting student accomplishment categories, i.e. grades or pass/fail, etc. The combined representation (*H*_*Combined*_) is then passed to fully connected layers, and a softmax activation generates the probability distribution across classes. Thus, the model combines local pattern recognition of LSTM and global context understanding of DistillBERT, providing an accurate predictive framework for academic success. The combined taxonomical organization [25] (proposition three citations) is illustrated in Eq.
  $$\begin{array}{c} SP_{pred}=SoftmaxAct\left(FullyConnecLayers\left(H_{Combined}\right)\right) \end{array}$$ (8)

**Fig 4:**
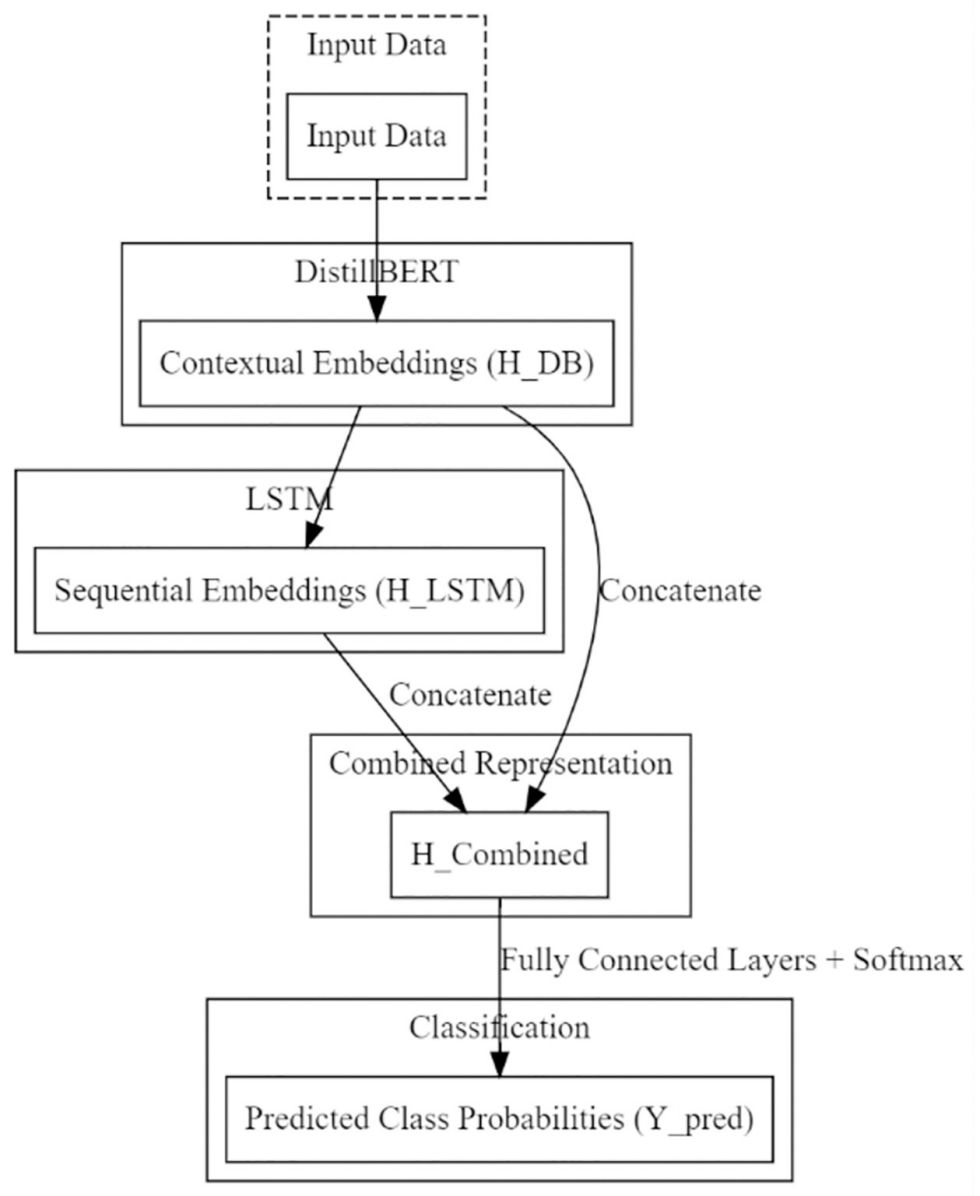
Proposed DBTM architecture of data flow.

The model improves its accuracy in predicting student achievement using an integrated approach, using local sequential information and global context. Fig 4 shows the DBMS framework.

### Tuning with SHO

This tuning approach acquires the Spotted Hyena Optimizer (SHO) [26] to enhance parameters (*ϕ*) & of DistillBERT with LSTM (DBTM) model, therefore enhancing its prediction on higher education student success forecasting as well. Based on some parameter settings specified by the objective function *F*(*ϕ*), called pack of hyenas and named pack*P*(0), this is introduced into the optimization process. This function includes key performance metrics tracked at different epochs in the model training, i.e., recall, precision, and F1-S score.

The hyena pack is adept at hunting and exploration tasks as each iterative cycle progresses. Each hyena has a different set of parameters evaluated for the fitness function. Cooperation and interaction between hyenas can ensure easy study of many prospective offers. The population update is based on evolutionary principles; it aims to keep those configurations with higher fitness alive, thus moving the pack of hyenas closer and closer to an optimal configuration. This collecting knowledge of pack hyenas is utilized to modify a DBTM setting. This series of adaptations and adjustments is simply a process called;

$$\begin{array}{c} \phi (t+1)=UpdateParameters(\phi (t),P(t+1)) \end{array}$$ (9)

Changes in learning rates, dropout rates, and other critical factors are utilized to develop the model’s structure. One essential property of SHO is its ability to respond to changes in data. The DBTM model is more adaptable to changes in student performance data due to the ability of its changed parameters, making it scalable. This iterative optimization loop refines parameter settings, leading to the optimum set (*ϕ*) that maximizes the fitness function. Algorithm 1: Tuning DBTM using SHO.

**Algorithm 1** Tuning using SHO

**Input:** Clean Data from Feature Engineering Step

**Objective Function:** *F*(*ψ*) representing the fitness of DBTM model parameters.

**Initial Pack of Hyenas:** *H*^(0)^ with diverse parameter configurations.

**Maximum Number of Iterations:** *T*.

**Output:** Optimal set of parameters: *ψ*.

1: **Algorithm Steps:**

2: **Initialization:** Release a pack of hyenas, each embodying a distinct parameter configuration: *H*^(0)^← ReleaseHyenaPack()

3: Set the iteration counter *k* = 1.

4: **Optimization Loop:** While *k* ≤ *T* do:

5:  a. **Hunting Phase:** Evaluate the fitness of each hyena’s parameter configuration:

6:   Evaluate Fitness: *F*(*ψ*^(*k*)^)

7:  b. **Update Hyena Pack:** Update the hyena pack based on the fitness values:

8:   Update Hyena Pack: *H*^(*k*+1)^ ← UpdateHyenaPack(*H*^(*k*)^, *F*)

9:  c. **Exploration:** Facilitate the exploration of novel parameter configurations through hyena collaboration and communication.

10:  d. **Update Parameters:** Update the DBTM model parameters based on hyena collaboration:

11:   Update Parameters: *ψ*^(*k*+1)^ ← UpdateParameters(*ψ*^(*k*)^, *H*^(*k*+1)^)

12: **Increment Iteration Counter:** *k* ← *k* + 1

13: **Result:** *ψ* ← arg max_*ψ*_ *F*(*ψ*)

**Fig 5:**
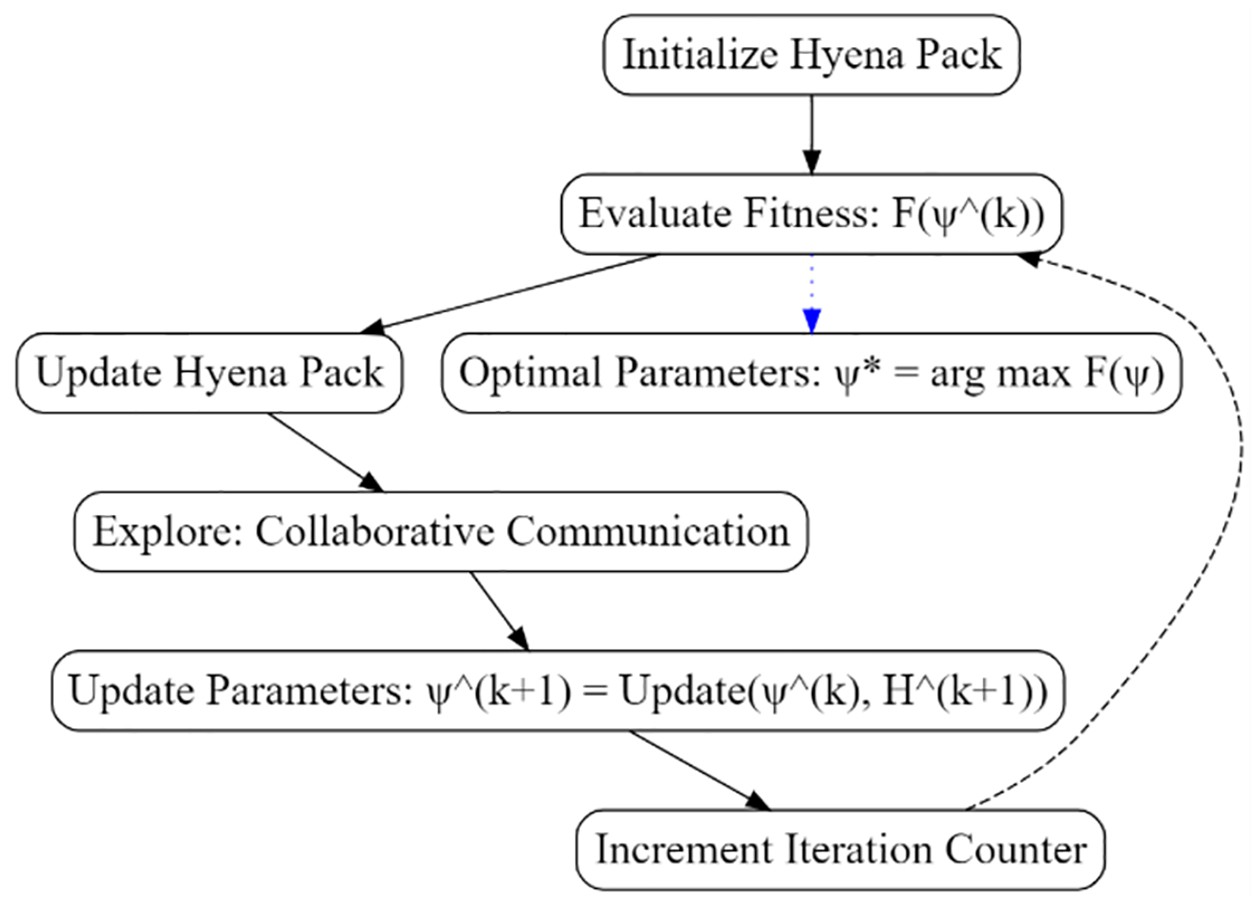
Parameter tuning process.

Adding an attribute configuration that materially increases the prediction power of the DBTM model. These rationalizations have been confirmed by thorough investigation and analysis using various data sets across different studies, among which study11 has further supported the updated DBTM model in real-life settings. Meanwhile, the analytical and prescriptive simulation framework associated with predicting academic success in kids is improved practically due to its simple optimization parameter process, which Spotted Hyena Optimizer wanted. Fig 5 describes the tuning process.

### Performance assessment

**Fig 6:**
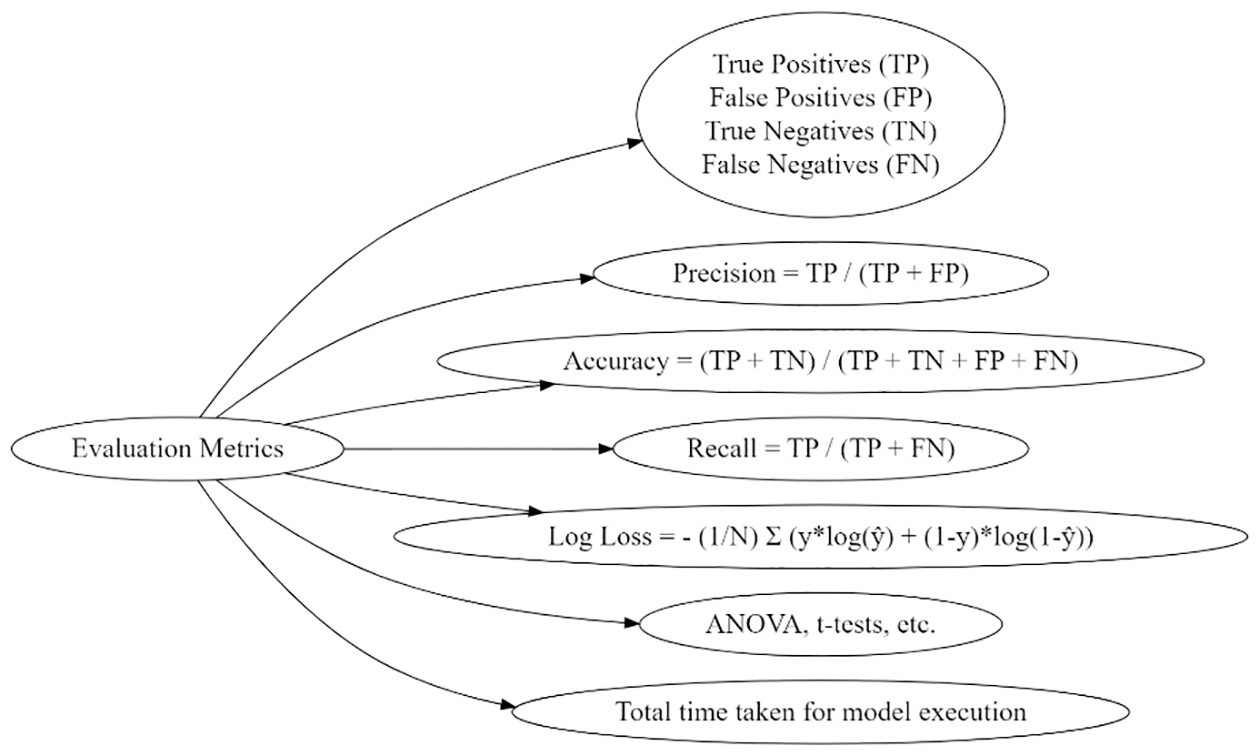
Performance metrics.

The efficacy of the proposed hybrid technique is evaluated using several classification measures, such as the confusion matrix, log loss, statistical analysis, and execution time. Using these assessment indicators, we analyze the effectiveness of categorization algorithms. The techniques are illustrated in Fig 6.

## Simulation and results

This study aims to improve the precision of predicting student performance by utilizing a machine with an Intel Core i7 11th Gen CPU operating at 2.4 GHz and coupled with a 4GB RTC graphics card. Python is the main language to run simulations in the Anaconda Spyder IDE.

The first stage is importing the dataset into the framework and preparing it for preprocessing. The dataset contains missing values, which are resolved using two independent approaches. Any row with more than 50% missing values is excluded. Alternatively, when the number of missing values in a row is below 50.

**Fig 7:**
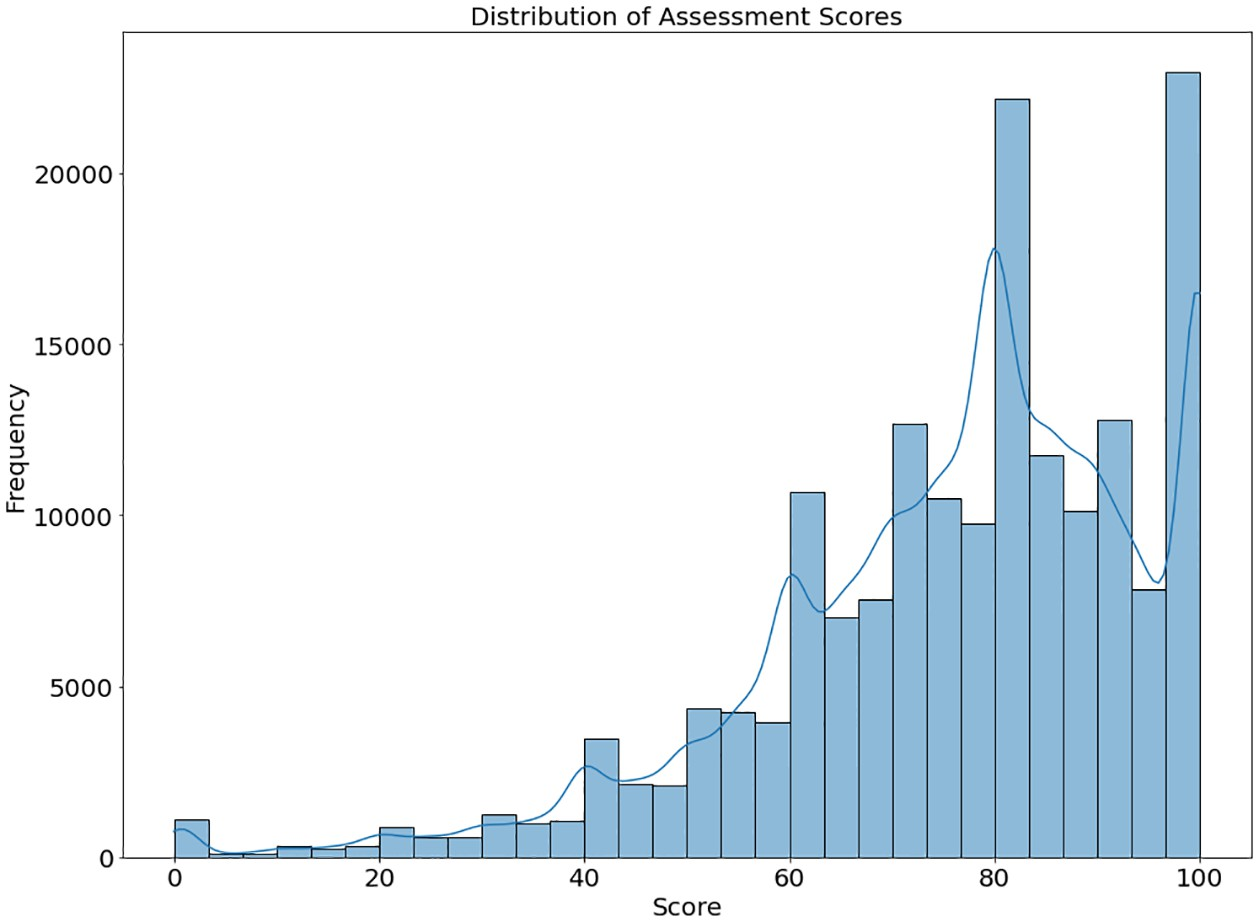
Distribution of dataset (Assessment score).

Exploratory Data Analysis (EDA) is conducted on the dataset to acquire insights and understand the data. Fig 7 displays the histogram representing the variability of assessment results obtained from the OULAD dataset. Score ranges are shown on the x-axis, and the frequency of occurrences within each range is shown on the y-axis. A kernel density estimate (KDE) curve is superimposed over the distribution to offer a normalized representation of the data distribution. The histogram clearly shows that most students’ results are concentrated within specified intervals, as the conspicuous peaks show. The KDE curve facilitates the identification of any underlying trends or patterns in the assessment scores. This graphical representation facilitates the rapid assessment of outliers and core tendencies. The distribution’s tails can indicate the presence of exceptionally high or low scores.

**Fig 8:**
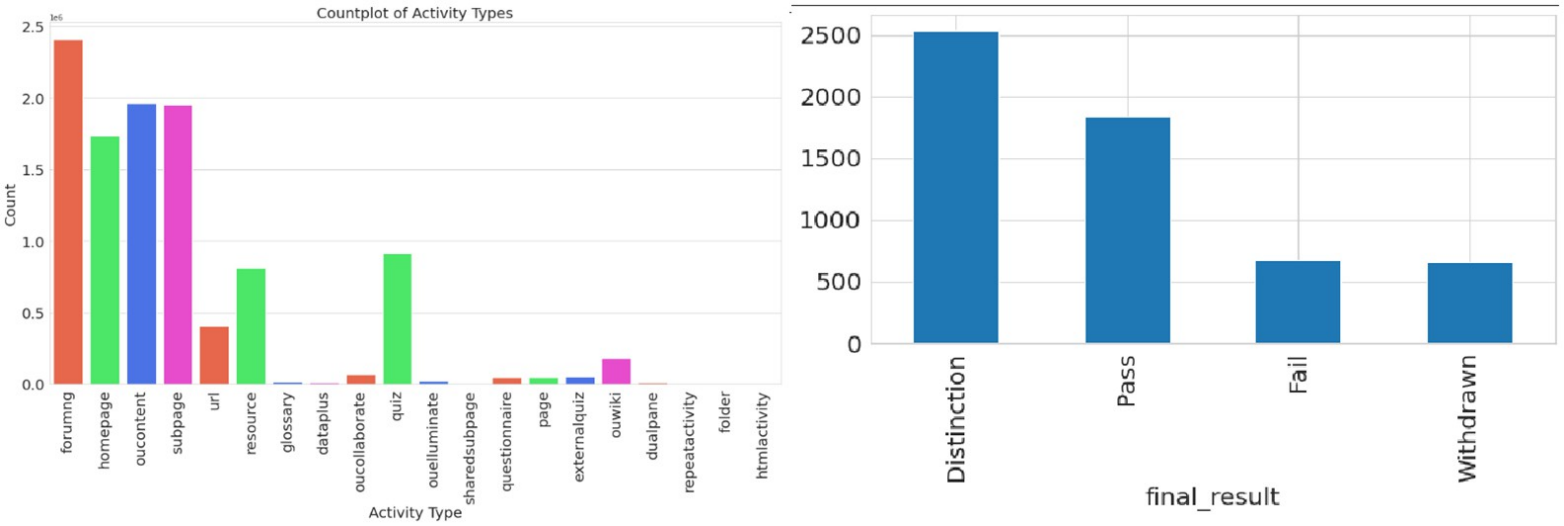
Activity types and final result.

The relationship between student involvement and academic results is well shown in Fig 8. A count plot of distinct VLE activity categories is shown on the left side of the figure, illustrating the varied degrees of student participation. A unique color palette makes it simpler to differentiate between different activities, making it easier to identify engagement patterns and how frequently each type is utilized. Conversely, the main focus of the figure on the right is the average number of interactions (after clicks) according to students’ final findings. Students with better grades, particularly those who get distinctions, use the virtual learning environment (VLE) considerably more than their peers who do worse. Taken as a whole, these numbers show how vital participation is for students to do well in school and illuminate the connections between various VLE activities and grades.

**Fig 9:**
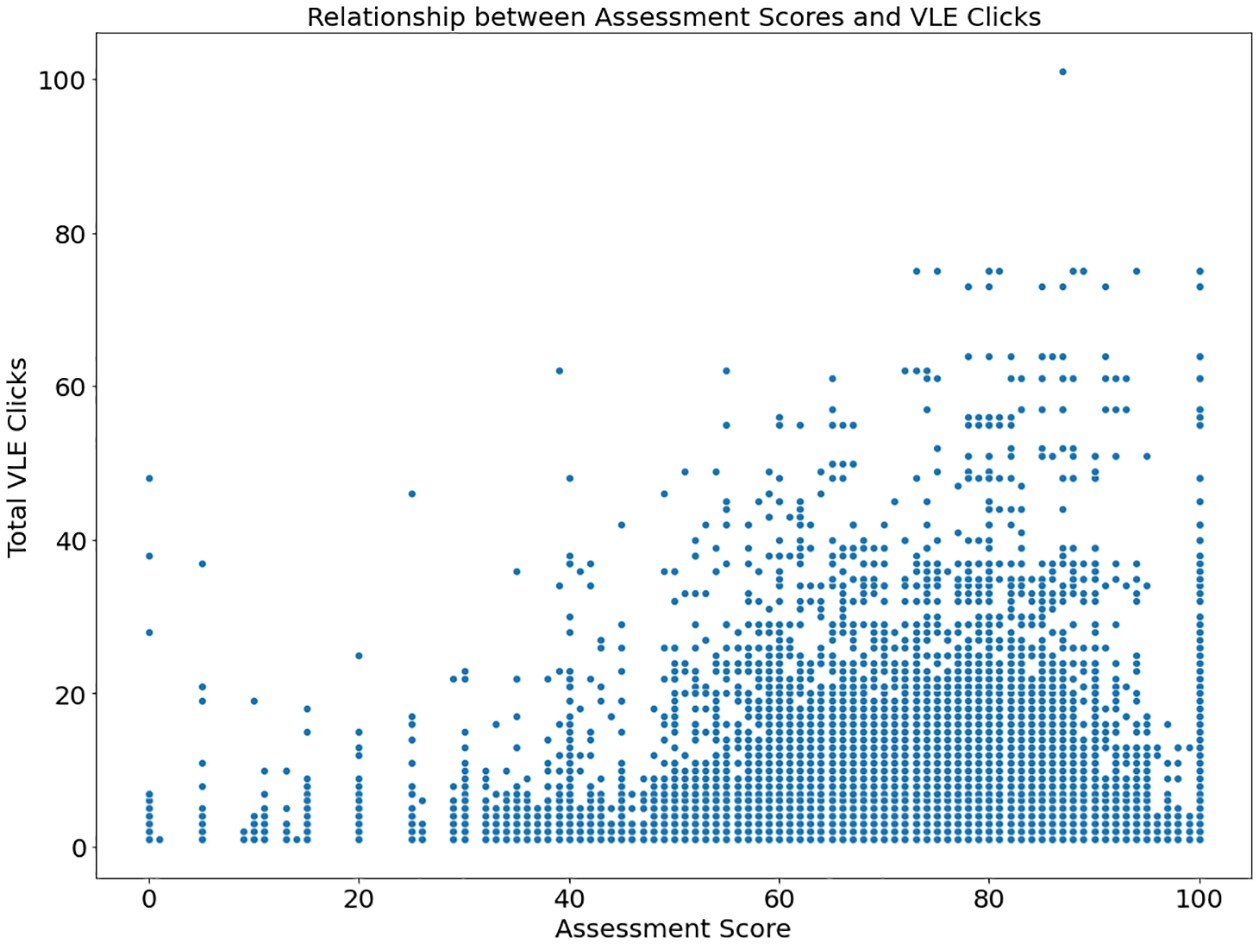
Distribution of dataset (Relation between score and VLE).

Fig 9 explains how students’ participation in the VLE and their assessment results relate. As a measure student’s academic achievement, the x-axis displays assessment results. In contrast, the total quantity of clicks pupils performed in the VLE is displayed on the y-axis, measuring their online activity.

Patterns and trends within the data can be observed in Fig 9. A positive correlation between assessment scores and Virtual Learning Environment (VLE) clicks is suggested if the graph’s points demonstrate an increasing trend from left to right, learners actively participating in the virtual classroom typically achieve higher test scores. Conversely, a lack of clear trends or discrepancies among points indicates a weak or nonexistent relationship between VLE engagement and academic performance.

**Fig 10:**
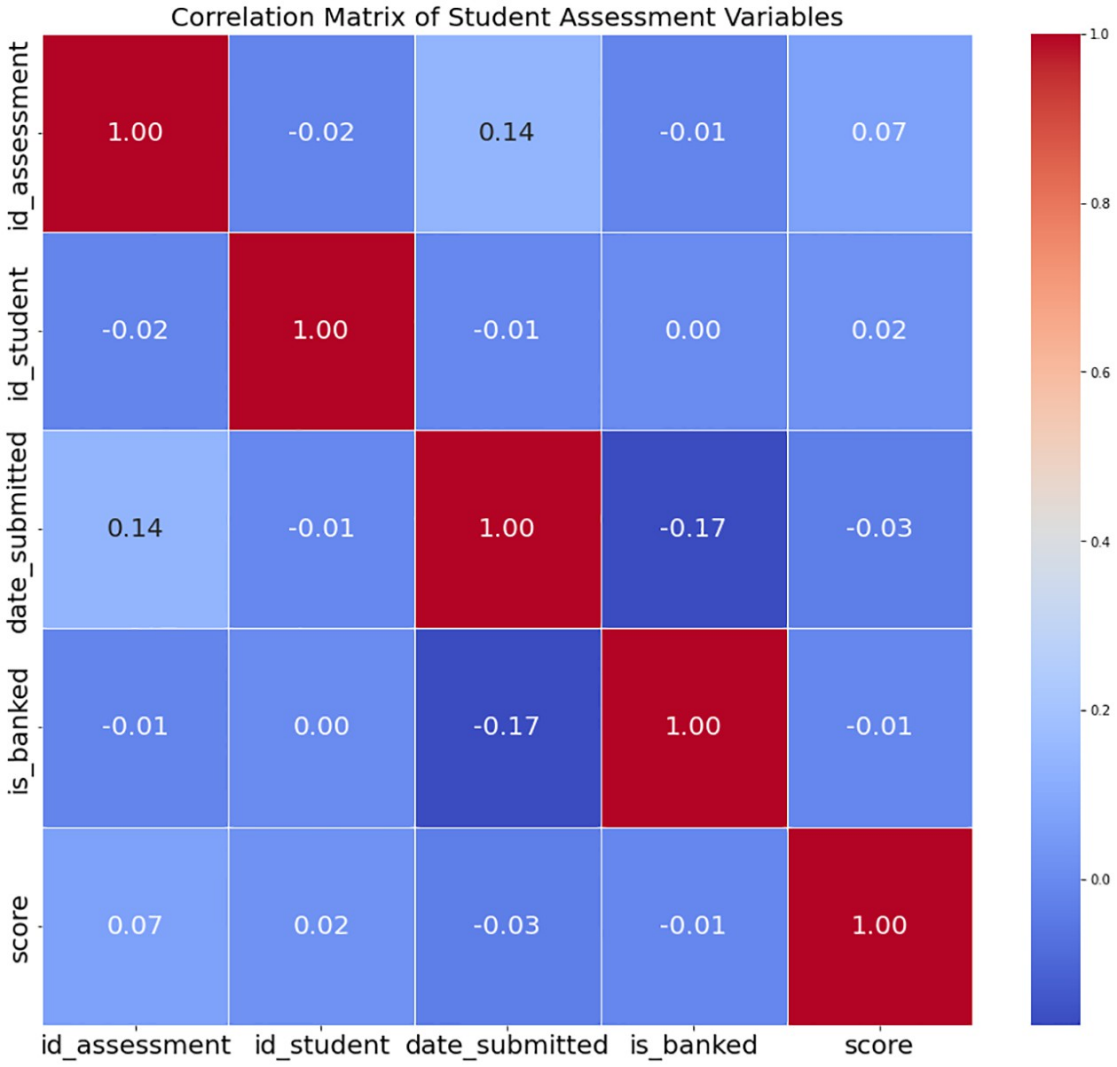
Numeric attributes correlation.

Fig 10 shows the dataset’s component correlation matrix, which provides useful information about the correlations between different assessment measures. On the heatmap, different colors represent different degrees of association. Lighter shades show weak correlations, dark red shows strong positive correlations, and dark blue shows major negative correlations. The numerical annotations within each heatmap cell represent specific correlation coefficients ranging from -1 to 1. By interpreting these coefficients, readers can discern trends and associations between pairs of variables. Negative coefficients suggest an inverse relationship, while positive coefficients indicate variables that tend to move in tandem. The heatmap facilitates the identification of clusters of factors with strong correlations, guiding further research efforts.

In Table 3, we present a comprehensive performance evaluation of our proposed method alongside existing approaches documented in the literature. The results are extensively examined, compared to those obtained by other researchers, using numerous methodologies. A DBTM-SHO performs well in all metrics, making it visible from a performance evaluation perspective. These numbers indicate how effective our architecture is and what its scale can be in the industry.

**Table 3:** Analyzing the new method’s performance in contrast to the current one.

Metric	DBTM-SHO	SVM [16]	NN [17]	DT [19]	NB [25]	LR [27]	ResNet [28]
Accuracy	0.9898	0.8563	0.8771	0.8624	0.8241	0.8174	0.8743
Kappa statistic	0.9839	0.8562	0.8815	0.8685	0.8378	0.8243	0.8781
Precision	0.9872	0.8745	0.8832	0.8661	0.8492	0.8325	0.8713
AP	0.9861	0.8774	0.8992	0.8698	0.8561	0.8456	0.8875
F1 Score	0.984	0.8652	0.8825	0.8678	0.8387	0.8237	0.8795
Log Loss	0.0379	0.3682	0.3288	0.3513	0.3958	0.4165	0.3462
Recall	0.9829	0.8579	0.8855	0.8645	0.8311	0.8294	0.8716
MCC	0.986	0.8537	0.8841	0.8653	0.8342	0.8215	0.8722
G-mean	0.9934	0.9041	0.9267	0.9036	0.8834	0.8782	0.9223
Specificity	0.9848	0.9026	0.9354	0.9142	0.8951	0.8705	0.9236

A comparison of the categorization approach proposed in this research with others is shown, which can be seen in Table 4. Understanding how valid and valuable the results of this thorough investigation are. Table findings display positive benefits across all research variables, which validates our strategy as being hardy. This detailed statistical analysis validates the performance obtained by our method and, more importantly, sheds light on the architecture underlying categorization. The positive correlations among the variables help confirm that our classification algorithms are authentic and valuable, maybe even practical under real-world use.

**Table 4:** Statistical analysis results on the OULAD dataset.

Techniques	Chi-Squared	Spearman’s	Wilcoxon	Paired Student	Student	ANOVA
DBTM-SHO (F-stat)	174	0.832	7.642	0.05	0.01	49.315
DBTM-SHO (P-val)	0	0.015	0.032	0.002	0.07	0.001
SVM (F-stat)	215	0.674	6.72	-4.765	-1.904	56.987
SVM (P-val)	0	0.029	0.041	0.001	0.041	0.002
NN (F-stat)	192	0.745	7.219	-4.123	-1.632	51.763
NN (P-val)	0	0.022	0.029	0.001	0.054	0.001
DT(P-val)	180	0.81	7.923	-3.895	-1.345	47.924
DT(Fstat)	0	0.018	0.037	0.001	0.065	0.001
NB (F-stat)	286	0.532	5.983	-5.315	-2.47	62.183
NB (P-val)	0	0.043	0.049	0	0.036	0.001
LR(F-stat)	240	0.612	6.385	-4.981	-2.089	58.42
LR (P-val)	0	0.036	0.042	0.001	0.049	0.002
ResNet(F-stat)	326	0.458	5.32	-6.239	-2.987	68.925
ResNet(P-val)	0	0.054	0.055	0	0.027	0.001

**Fig 11:**
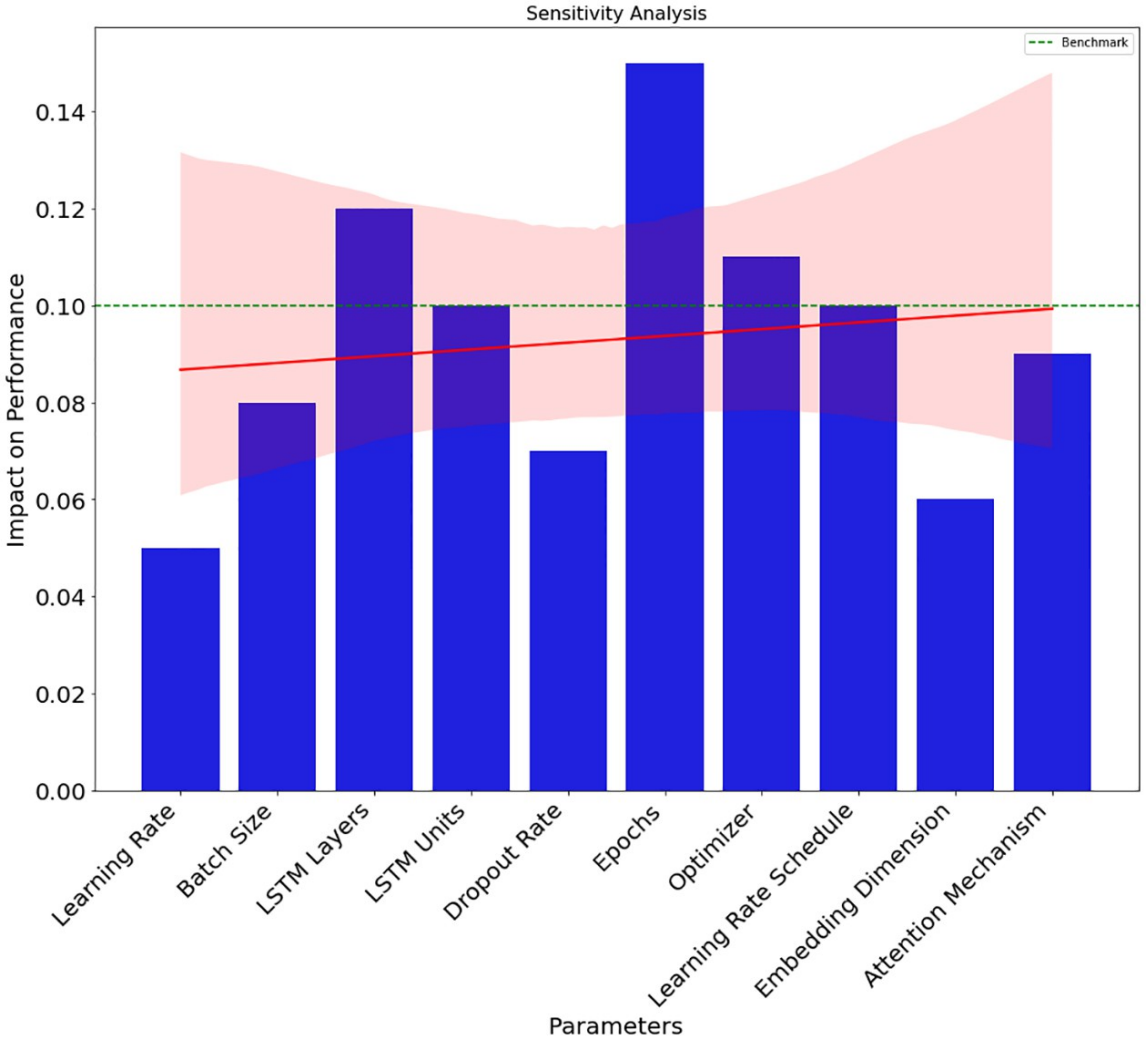
Sensitivity study to determine how factors affect model performance.

Fig 11 depicts a sensitivity study that investigates the impact of various factors on the model’s efficacy. The effect of each parameter on the model’s overall performance may be seen clearly in the bar chart. A red trend line shows the overall trend in the parameters’ effects, and each bar’s height shows the trend’s size. If the parameters are above the benchmark line (the dotted green line at 0.1), then the effect is above average; if they are below, then the influence is below average. There is a numerical description of the influence of each parameter next to each bar, which represents that parameter. This sensitivity analysis may assist researchers and practitioners in making informed decisions during model optimization by illuminating elements that significantly affect model accuracy.

**Fig 12:**
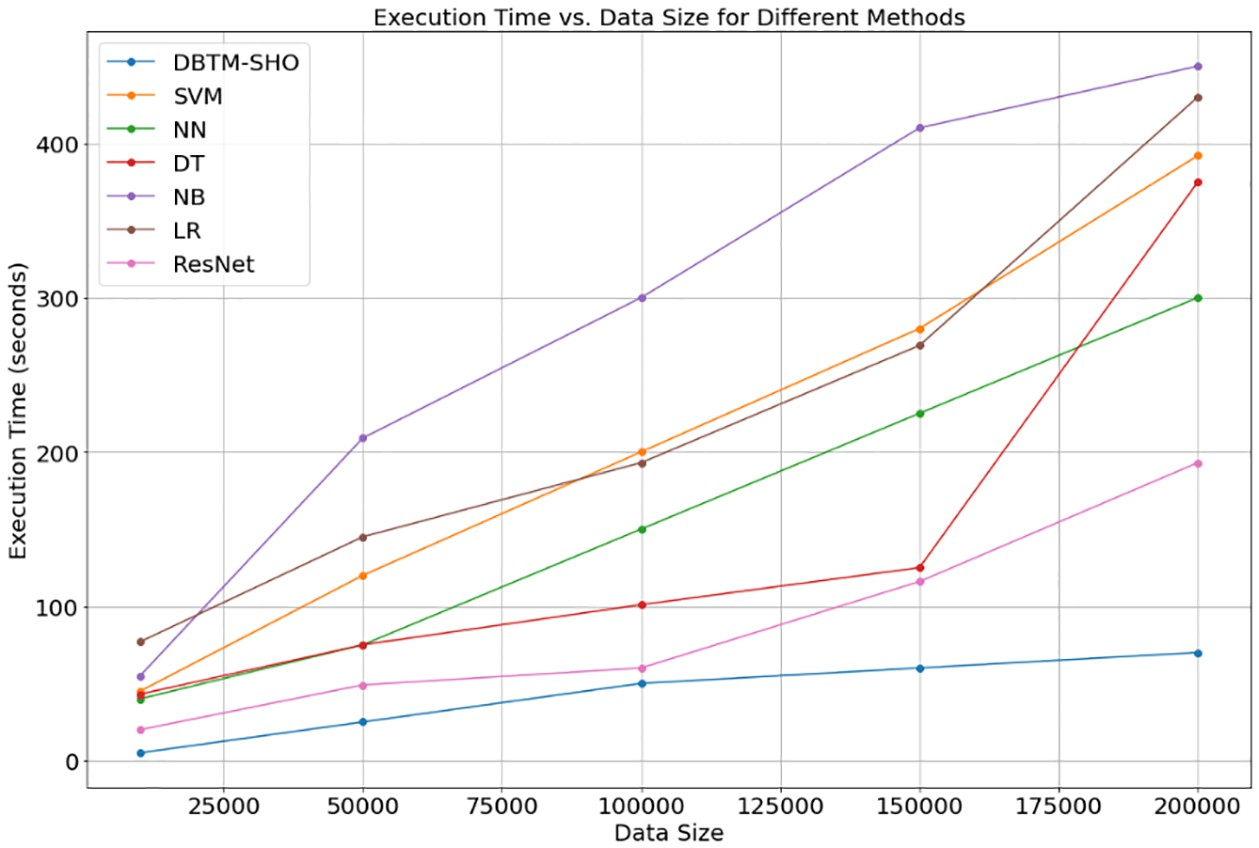
Connection between data amount and duration of execution.

Fig 12 shows the proposed DBTM-SHO technology and other existing methods as a function of data size and execution time. By plotting execution times in seconds against data sizes on the x-axis and 10,000 to 200,000 on the y-axis, we can see the relationship between the two variables. Markers on the graph indicate the amount of time required to execute for different data amounts, and each line on the graph indicates a different approach. As represented by the figure, the execution time could vary significantly depending on the approach and the dataset size. Database Transform SHO’s execution time remains relatively consistent as the volume of data increases. The processing efficacy of SVM and NB is more notable when the datasets are larger. The distinct patterns of execution time that NN, DT, LR, and ResNet display in response to data size demonstrate that various approaches have varied processing demands and scalability characteristics.

## Conclusion

The results of this study demonstrate that educational institutions can benefit from utilizing enhanced ML algorithms to predict how well their students will do in the future. Significant enhancements in accuracy, decrease in log loss, and improvement in execution time have been accomplished by building a comprehensive framework that unifies data preparation, feature engineering, and classification utilizing the hybrid DBTM-SHO model. Demonstrating its capacity to efficiently manage expanding volumes of data in graduate and postgraduate programs, the model achieves noteworthy performance metrics—98.7% accuracy, 0.03% log loss, and 15%–25% optimized execution time—or more. The findings underscore the model’s potential as a valuable instrument for examining educational data. The consequences of these discoveries are significant for all those involved in education. The methodology that has been suggested establishes a robust framework for the timely identification of children who might be at risk, thereby facilitating tailored support strategies and interventions. Through the implementation of predictive analytics, educators and administrators have the potential to improve student outcomes, maximize the efficiency of current resources, and elevate the overall efficacy of the educational system. In addition, the amalgamation of optimization algorithms and cutting-edge machine learning methodologies streamlines the decision-making process based on data. It facilitates individualized learning experiences, thereby augmenting student achievement.

## Limitations and future research directions

The study’s findings are encouraging; however, some constraints must be addressed to make the model more applicable and generalizable. For example, the model may not apply to other educational settings due to its exclusivity to the OULAD dataset; these settings and student demographics may vary greatly. Future research should assess this approach utilizing many datasets from various institutions to ensure its durability and flexibility. Second, the objective of this work is to predict student performance with structured data that contains demographic information and module-related algorithms, as well as public software for interaction within the virtual classroom. The model can be improved further by adding more unstructured data sources such as forum entries, textual notes, etc. Applying NLP techniques to text data would provide a fuller picture of students’ learning behaviors and performance predictors. In addition, the computational overhead and resource requirements of the proposed hybrid model (SHO-DBTM) must be considered. Future work should focus on optimizing model performance without impairing prediction. Model reduction, feature selection, and distributed computing architectures effectively address these challenges; consequently, the final model is more suited for educational online applications.
